# Radiation treatment of benign tumors in NF2-related-schwannomatosis: A national study of 266 irradiated patients showing a significant increase in malignancy/malignant progression

**DOI:** 10.1093/noajnl/vdad025

**Published:** 2023-03-11

**Authors:** D Gareth Evans, Dorothy Halliday, Rupert Obholzer, Shazia Afridi, Claire Forde, Scott A Rutherford, Charlotte Hammerbeck-Ward, Simon K Lloyd, Simon M Freeman, Omar N Pathmanaban, Owen M Thomas, Roger D Laitt, Stavros Stivaros, John-Paul Kilday, Grace Vassallo, Catherine McBain, Timothy Lavin, Chay Paterson, Gillian Whitfield, Martin G McCabe, Patrick R Axon, Jane Halliday, Samuel Mackeith, Allyson Parry, English Specialist NF2 Research Group, Patrick Axon, Juliette Buttimore, James Tysome, Neil Donnelly, Daniele Borsetto, James Whitworth, Anke Hensiek, Rajesh Jena, Mathew Guilfoyle, Richard Mannion, James Nicholson, Brinda Muthusamy, Amy Taylor, Richard Price, Karine Edme, Nicola Gamazo, Zebunnisa Vanat, Daniel Scoffings, Josh Scott, Sarah Jefferies, Richard Knight, Tamara Lamb, Yu Chuen Tam, Karen Foweraker, Fiona Harris, Paul Sanghera, Sara Meade, Richard Irving, Peter Monksfield, Nicola Ragge, Melanie Murrell, Julian Barwell, Martin English, Rikin Trivedi, Shazia K Afridi, Rosalie E Ferner, Rupert Obholzer, Victoria Williams, Chris Hammond, Karine Lascelles, Chris Skilbeck, Adam Shaw, Angela Swampillai, Suki Thomson, Nick Thomas, Eleni Maratos, Sinan Barazi, Rebecca Mullin, Susie Henley, Natalie Smith, Lal Carlton-Jones, Alison Baker, Mandy Myers, Terry Nunn, Charles Nduka, Raji Anup, Chris Duff, Simon R Freeman, Nicola Jarvis, Ian Kamaly-Asl, Andrew T King, Mark Kellett, John-Paul Kilday, Simon K Lloyd, Catherine McBain, Roger Laitt, Martin O’Driscoll, Martin McCabe, Mary Perry, Scott A Rutherford, Kirsty Henshaw, Stavros M Stivaros, Owen Thomas, Grace Vassallo, Charlotte L Hammerbeck-Ward, Omar N Pathmanaban, Jincy Kurian, Tim Lavin, Elaine F Harkness, Juliette Buttimore, Andrew T King

**Affiliations:** Division of Evolution and Genomic Medicine, Manchester Centre for Genomic Medicine, Manchester Academic Health Science Centre, University of Manchester, St Mary’s Hospital, Manchester Universities NHS Foundation Trust, Manchester, UK; Geoffrey Jefferson Brain Research Centre, Manchester Academic Health Science Centre, Northern Care Alliance NHS Foundation Trust, University of Manchester, UK; Departments of Genomic Medicine, Neurology, Neurosurgery, ENT, Oxford University Hospitals NHS Foundation Trust; Department of ENT, and Neurology, London, UK; Guy’s and St Thomas’ NHS Trust, London, UK; Division of Evolution and Genomic Medicine, Manchester Centre for Genomic Medicine, Manchester Academic Health Science Centre, University of Manchester, St Mary’s Hospital, Manchester Universities NHS Foundation Trust, Manchester, UK; Departments of Neurosurgery, Otolaryngology, Neuroradiology, and Neurology, Manchester Centre for Clinical Neurosciences Salford Royal Hospital, Northern Care Alliance NHS Foundation Trust, Manchester, UK; Departments of Neurosurgery, Otolaryngology, Neuroradiology, and Neurology, Manchester Centre for Clinical Neurosciences Salford Royal Hospital, Northern Care Alliance NHS Foundation Trust, Manchester, UK; Departments of Neurosurgery, Otolaryngology, Neuroradiology, and Neurology, Manchester Centre for Clinical Neurosciences Salford Royal Hospital, Northern Care Alliance NHS Foundation Trust, Manchester, UK; Departments of Neurosurgery, Otolaryngology, Neuroradiology, and Neurology, Manchester Centre for Clinical Neurosciences Salford Royal Hospital, Northern Care Alliance NHS Foundation Trust, Manchester, UK; Departments of Neurosurgery, Otolaryngology, Neuroradiology, and Neurology, Manchester Centre for Clinical Neurosciences Salford Royal Hospital, Northern Care Alliance NHS Foundation Trust, Manchester, UK; Division of Neuroscience and Experimental Psychology, Faculty of Biology, Medicine and Health, University of Manchester, UK; Geoffrey Jefferson Brain Research Centre, Manchester Academic Health Science Centre, Northern Care Alliance NHS Foundation Trust, University of Manchester, UK; Departments of Neurosurgery, Otolaryngology, Neuroradiology, and Neurology, Manchester Centre for Clinical Neurosciences Salford Royal Hospital, Northern Care Alliance NHS Foundation Trust, Manchester, UK; Departments of Neurosurgery, Otolaryngology, Neuroradiology, and Neurology, Manchester Centre for Clinical Neurosciences Salford Royal Hospital, Northern Care Alliance NHS Foundation Trust, Manchester, UK; Division of Informatics, Imaging and Data Sciences, School of Health Sciences, Faculty of Biology, Medicine and Health, The University of Manchester, Manchester Academic Health Science Centre, Oxford Road, Manchester, M13 9PL, UK; Academic Unit of Paediatric Radiology, Royal Manchester Children’s Hospital, Central Manchester University Hospitals NHS Foundation Trust, Manchester Academic Health Science Centre, Manchester, UK; Geoffrey Jefferson Brain Research Centre, Manchester Academic Health Science Centre, Northern Care Alliance NHS Foundation Trust, University of Manchester, UK; Children’s Brain Tumour Research Network (CBTRN), Royal Manchester Children’s Hospital, Manchester, UK; Division of Cancer Sciences, Faculty of Biology, Medicine and Health, University of Manchester, UK; Division of Evolution and Genomic Medicine, Manchester Centre for Genomic Medicine, Manchester Academic Health Science Centre, University of Manchester, St Mary’s Hospital, Manchester Universities NHS Foundation Trust, Manchester, UK; The Christie NHS Foundation Trust, Manchester, UK; Geoffrey Jefferson Brain Research Centre, Manchester Academic Health Science Centre, Northern Care Alliance NHS Foundation Trust, University of Manchester, UK; Departments of Neurosurgery, Otolaryngology, Neuroradiology, and Neurology, Manchester Centre for Clinical Neurosciences Salford Royal Hospital, Northern Care Alliance NHS Foundation Trust, Manchester, UK; Division of Evolution and Genomic Medicine, Manchester Centre for Genomic Medicine, Manchester Academic Health Science Centre, University of Manchester, St Mary’s Hospital, Manchester Universities NHS Foundation Trust, Manchester, UK; Children’s Brain Tumour Research Network (CBTRN), Royal Manchester Children’s Hospital, Manchester, UK; Division of Cancer Sciences, Faculty of Biology, Medicine and Health, University of Manchester, UK; The Christie NHS Foundation Trust, Manchester, UK; Division of Cancer Sciences, Faculty of Biology, Medicine and Health, University of Manchester, UK; The Christie NHS Foundation Trust, Manchester, UK; Department of Otolaryngology, Cambridge University Hospitals NHS Foundation Trust, Cambridge, UK; Departments of Genomic Medicine, Neurology, Neurosurgery, ENT, Oxford University Hospitals NHS Foundation Trust; Departments of Genomic Medicine, Neurology, Neurosurgery, ENT, Oxford University Hospitals NHS Foundation Trust; Departments of Genomic Medicine, Neurology, Neurosurgery, ENT, Oxford University Hospitals NHS Foundation Trust; Division of Informatics, Imaging and Data Sciences, School of Health Sciences, Faculty of Biology, Medicine and Health, The University of Manchester, Manchester Academic Health Science Centre, Oxford Road, Manchester, M13 9PL, UK; Prevent Breast Cancer Centre, Wythenshawe Hospital, Manchester Universities NHS Foundation Trust, Manchester, UK; Department of Otolaryngology, Cambridge University Hospitals NHS Foundation Trust, Cambridge, UK; Departments of Neurosurgery, Otolaryngology, Neuroradiology, and Neurology, Manchester Centre for Clinical Neurosciences Salford Royal Hospital, Northern Care Alliance NHS Foundation Trust, Manchester, UK; Geoffrey Jefferson Brain Research Centre, Manchester Academic Health Science Centre, Northern Care Alliance NHS Foundation Trust, University of Manchester, UK; Division of Cardiovascular Sciences, Faculty of Biology, Medicine and Health, University of Manchester, UK

**Keywords:** malignant transformation, MPNST, NF2, Radiotherapy, schwannoma

## Abstract

**Background:**

Radiation treatment of benign tumors in tumor predisposition syndromes is controversial, but short-term studies from treatment centers suggest safety despite apparent radiation-associated malignancy being reported. We determined whether radiation treatment in NF2-related schwannomatosis patients is associated with increased rates of subsequent malignancy (M)/malignant progression (MP).

**Methods:**

All UK patients with NF2 were eligible if they had a clinical/molecular diagnosis. Cases were NF2 patients treated with radiation for benign tumors. Controls were matched for treatment location with surgical/medical treatments based on age and year of treatment. Prospective data collection began in 1990 with addition of retrospective cases in 1969. Kaplan–Meier analysis was performed for malignancy incidence and survival. Outcomes were central nervous system (CNS) M/MP (2cm annualized diameter growth) and survival from index tumor treatment.

**Results:**

In total, 1345 NF2 patients, 266 (133-Male) underwent radiation treatments between 1969 and 2021 with median first radiotherapy age of 32.9 (IQR = 22.4–46.0). Nine subsequent CNS malignancies/MPs were identified in cases with only 4 in 1079 untreated (*P* < .001). Lifetime and 20-year CNS M/MP was ~6% in all irradiated patients—(4.9% for vestibular schwannomas [VS] radiotherapy) versus <1% in the non-irradiated population (*P* < .001/.01). Controls were well matched for age at NF2 diagnosis and treatment (Males = 133%–50%) and had no M/MP in the CNS post-index tumor treatment (*P* = .0016). Thirty-year survival from index tumor treatment was 45.62% (95% CI = 34.0–56.5) for cases and 66.4% (57.3–74.0) for controls (*P* = .02), but was nonsignificantly worse for VS radiotherapy.

**Conclusion:**

NF2 patients should not be offered radiotherapy as first-line treatment of benign tumors and should be given a frank discussion of the potential 5% excess absolute risk of M/MP.

**Key Points**

- Radiotherapy is associated with an increased risk of malignancy in NF2.
- Survival in matched controls is worse after radiotherapy in NF2.

**Importance of the Study**

Patients with NF2 are increasingly being offered radiation treatments as first-line therapy for vestibular schwannoma and meningioma and the risk of malignancy is usually downplayed as there is currently no reliable data on actual risk. The current study encompasses real-world data from 266 NF2 patients who have received radiotherapy for benign tumors over a more than 50-year time period. The study is a total country audit with very high patient ascertainment and completes follow-up to death. The study shows a 6% 20-year risk of CNS malignancy/malignant progression in radiation-treated patients compared to <1% in non-irradiated patients. The study provides, for the first time, an accurate assessment of malignancy risk that can be used when discussing radiation treatment with NF2 patients. The study also has implications for malignancy in other tumor predisposition syndromes, beyond NF1, where the malignancy risk is already established.

Neurofibromatosis type 2, now called NF2-related-schwannomatosis,^1^ is an autosomal dominant tumor predisposition disorder with high penetrance and reduced life expectancy.^2–4^ The predisposition is primarily to benign nerve sheath tumors (schwannomas), benign meningiomas, and usually indolent slow-growing spinal ependymomas.^2,5^ True CNS malignancy in the absence of radiation treatment is very rare.^5,6^ Despite a number of reports indicating apparent safety in regards to radiation treatments in NF2,^7–10^ there remain concerns about malignant induction risk.^11^ Selection of NF2 patients for radiation treatment is complex and balances patient choice with age of the patient, requirement for hearing preservation particularly on the second side VS and also for treatment of residual tumors after surgery. Whilst the majority of clinical reports refer to stereotactic radiation treatment for vestibular schwannomas (VS),^7–9^ meningioma treatments with radiation have also become more common recently.^10^ All reports are limited by the number of NF2 patients treated and relatively short follow-up. Although malignant transformation of schwannomas post-irradiation is uncommon it is noteworthy that almost half the cases following VS radiation treatments occurred in NF2 patients whilst probably less than 5% of the cases treated were NF2 patients.^11^ There are concerns not just about the potential increased risks of malignant progression (MP) in the often, multifocal tumors of NF2 but also in the induction of new malignancies or indeed meningiomas and schwannomas in the radiation volume (field) in this tumor-predisposing condition.^12^

This work evaluates the whole UK experience of radiation treatments in NF2, representing over 30 years of prospective assessments, in a case–control methodology to assess malignancy risk and survival from treatment.

## Methods

Patients meeting clinical diagnostic criteria for NF2 and who had resided or were residing in the United Kingdom were eligible for assessment. The UK NF2 national database has been recording NF2 patients since 1990 and has close to complete ascertainment of all NF2 patients in the United Kingdom based on a nationally commissioned highly specialized service for England inaugurated in 2010.^2–4^ Patients meeting NF2 diagnostic criteria were excluded based on the presence of a germline *LZTR1* variant and/or different pathogenic variants in the *NF2* gene in anatomically distinct tumors in the absence of germline or common PV in tumors (*n* = 16) as well as cases born before 1900 (*n* = 3). Cases were defined as those who had undergone radiation treatment for a benign, or presumed benign, central nervous system (CNS) tumor hereafter called the index tumor. This included stereotactic (nearly all Gamma Knife) radiotherapy or fractionated treatments for a CNS schwannoma, meningioma, or ependymoma. A case–control or matched retrospective cohort study analysis was carried out by matching cases to controls within the NF2 national database who were within 3 years of age at the time of treatment of the relevant tumor type (vestibular schwannoma, cranial meningioma, or spinal tumor), this included second or subsequent treatments of tumors before the “index” tumor. Controls were then also matched on year of birth (ideally within 4 years), and where possible, for sex. Unfortunately, matching was not possible on tumor size as many cases were historic without available imaging, but the vast majority of VS >3.5cm had surgery, not radiation therapy. The usual limit agreed upon by the main radiosurgery center (Sheffield) in the United Kingdom is a 3cm diameter^13^ based on previous poor responses in these larger tumors.^14^ As all radiotherapy referrals for NF2 in the last 12 years have been from the 4 centers represented in this paper and the great majority in the 10 years prior to that we are confident that very few tumors exceeded 3.5cm in diameter at radiation treatment.

Follow-up was assessed from tumor treatment date (surgery or start of bevacizumab for controls) to date of last follow-up, death, or date of malignant tumor occurrence/progression. Whilst the great majority of radiation treatments were first treatments without prior surgery, second or third treatments for the controls were allowed to prevent bias. Where final histology was not available a 2cm annualized growth for schwannoma, meningioma, or ependymoma was considered MP. For schwannoma, a diagnosis from the contemporaneous histopathological report of malignant peripheral nerve sheath tumor (MPNST) or Triton tumor and for meningioma World Health Organization (WHO) grade 3 histology was considered malignant.^15^ WHO Grade 3 or above for glial tumors were considered malignant. MP of the treated tumor or a new onset CNS malignancy of any kind were included in both the irradiated and non-irradiated cases. Tumors were considered as potentially radiation-induced if they arose within the high or low dose radiation volumes (ie, within the radiation field including the beam penumbra), and not radiation-induced if they received no radiation or a negligible dose (ie, arose beyond the radiation field, receiving at most low dose scattered irradiation). Kaplan–Meier was used to assessing (1) cumulative incidence of MP/occurrence, and (2) cumulative survival from date of diagnosis to death. *P*-values of less than .05 were regarded as statistically significant. Analysis was performed in Stata version.v.14 (StataCorp. 2015. Stata Statistical Software: Release 14. College Station, TX: StataCorp LP).

Molecular analysis was performed in Manchester as previously described using both blood and where available tumor DNA.^16^ Latterly from 2013 the analysis included next-generation sequencing of the coding sequence and intron/exon boundaries as well as a test for large rearrangements with Multiple Ligation dependent Probe Amplification (MLPA).

Ethical approval for this study is covered by the North West – Greater Manchester Central Research Ethics Committee (reference 10/H1008/74).

## Results

A total of 1345 NF2 patients meeting clinical or molecular criteria and having resided in the United Kingdom were identified (Table 1). Two hundred and sixty-six patients (19.8%) had undergone radiation treatments for VS (*n* = 209 cases, 208 first treatment, 53 bilateral), cranial meningioma(s) (*n* = 49; 42 first treatments (VS treated first in 7), 10 multiple episodes) and spinal tumor(s) (*n* = 15). The earliest treatment was in 1969 and only 20/266 (7.5%) had their first treatment before 1990. The vast majority of the VS treatments were Gamma Knife with peripheral margin doses ranging from 10 Gy to 25 Gy (most treatments 12–15 Gy) and were carried out at the National Centre for Stereotactic Radiosurgery in Sheffield. Most meningioma treatments were also Gamma Knife but included multiple meningioma treatments simultaneously (maximum 5) and 10 had more than one episode of treatment, there were also 5 fractionated treatments. All spinal treatments except one were fractionated mainly to treat presumed intraspinal ependymoma. Four patients received proton beam therapy—1 spinal ependymoma, 3 meningiomas. Forty-seven patients received radiation treatment before age 20 (17.7%) and 32 (12.1%) before age 18. There were 304 patients from the full series that had died including 25 in the postoperative period: 20 in the non-irradiated group (1.9%) and a similar proportion (*n* = 5; 2.0%) in the irradiated group. Non-irradiated patients had a wider birth year distribution and fewer had a heterozygote PV identified (Table 2). Matching of cases and controls provided a very similar birth year and age at treatment distribution with non-irradiated cases having more confirmed heterozygote pathogenic variants and were slightly younger at index tumor treatment (Table 2). Bias is unlikely as time from diagnosis to index treatment was almost identical at 3.3 and 3.4 years and mean age at first symptom was also very close at 27.1 for non-irradiated versus 28.1 for irradiated.

**Table 1.** Number of Patients Undergoing Radiotherapy at Different Sites

	First Treatment	Radiation Dose	Second and Subsequent
*Single-fraction stereotactic treatments*			
Unilateral VS gamma knife/Cyberknife/Linac	187	Range 10–25Gy Median 14Gy IQR 12–16Gy	53*
Meningioma gamma knife single fraction	39	12–25Gy Median 15Gy IQR 12–15Gy	13
Trigeminal schwannoma gamma knife	1	12Gy	1
*Fractionated treatments*			
Unilateral VS photon	21	50–54Gy	
Spine photon	13	50–54Gy	1
Spine photon	1	50Gy	
Meningioma proton	3	50–54Gy	

**Table 2.** NF2 Patients Identified From UK Database, With and Without Radiation-Based Treatment

	No RT	No RT Matched	RT	*P*-value Matched Set
Number of patients with NF2	1079	266	266	
median age of NF2 diagnosis (years)	33.0	28.0	28.5	.98
IQR	18–50	19–43	18-44	
median year birth	1967	1971	1971	.95
IQR	1952–1986	1957–1983	1958–1981	
Mean age first symptom	28.3 (missing n=190)	27.07	28.10	
median age treatment index tumor (years)	-	31.3	32.9	.366
IQR	-	22.7-45.0	22.4-46.0	
Median time from NF2 diagnosis to treatment of index tumor (years)		1.61	2.75	
IQR		0.35–5.54	0.71–6.83	
Male	499	133	133	1.0
%	46.3%	50.0%	50.0%	
Follow-up from NF2 diagnosis years	12674.23	4580.54	4142.34	
Mean follow-up from NF2diagnosis (years)	11.75	17.22	15.57	.04
Median follow-up (years)	9.56	15.92	14.85	.13
IQR	5.1 – 16.9	9.3–24.0	8.9–22.1	
Follow-up from treatment of index tumor years	-	3778.12	2993.15	
Mean follow-up from treatment (years)	-	14.2	11.25	<.001
Median follow-up (years)	-	11.9	9.83	.005
IQR	-	5.8–20.0	4.1–16.6	
CNS malignancy from date of ­treatment (number)	5 (0.46%)	0	9* (3.4%)	.004
Sites RT/surgery	N/a	208 VS 42 meningioma 15 spinal	208 VS 42 meningioma 15 spinal	1.0
CNS malignancy types	2 epithelioid sarcomas 3 grade 3 meningioma	None -1 grade 3 meningioma but prior to matching	4 MPNST/malignant schwannoma 2 grade 3 meningioma 1 glioblastoma 2 aggressive ependymoma	
Died	232	58	72	.19
%	21.5%	21.8%	27.1%	
mean age death (years)	47.05	47.86	42.69	.07
Median (years)	44.68	45.79	39.75	.07
IQR	30.2–63.5	35.9–59.5	30.8–51.2	
CVA death	4	0	2	.50
% deaths	1.72%	0%	2.82%	
heterozygote PV	535	160	139	.08
% Heterozygote	49.58%	60.1%	52.3%	
Mosaic	189	63	49	.17
%	17.5%	23.7%	18.4%	
not found	355	43	78	.004
%	32.90%	16.2%	29.3%	
severe heterozygote PV	137	47	39	.28
%	12.7%	17.7%	14.7%	

RT-radiotherapy for benign/presumed benign disease; *-1 grade 3 meningioma excluded as occurred before RT and one MPNST, not in radiation volume; PV, pathogenic variant; IQR, interquartile range; VS, vestibular schwannoma; CVA, cerebrovascular accident (stroke).

Nine patients met the criteria for new malignancy (*n* = 7 pathology proven) or MP (*n* = 2) in the irradiated group (Table 3), and all but one qualified as potentially radiation-induced. A number have been previously reported including a glioblastoma 3 years post radiation in the brain stem between the bilateral VS radiations^7^ and a Triton tumor MP of a VS,^7^ although prior pathology was not available. MP of a VS was reported in a 2003 publication with clear evidence of initial benefit with shrinkage from radiotherapy and subsequent rapid growth that could not have been due to radiation time-specific edema or necrosis.^17^ A rapid ependymoma progression causing death (no pathology) and a grade 3 meningioma have also been previously reported.^18^ The current report contains 4 further new reports (Table 3): An MPNST in the VS 7 years after Gamma Knife, a MP of an ependymoma after spinal radiotherapy and a grade 3 meningioma which progressed rapidly over a 14-month period 16 years post-irradiation aged 15 years for a VS, with full treatment margins within 1cm of the subsequent initial meningioma formation. The final patient had a rapidly growing malignant nerve sheath tumor at L4, therefore not in the radiation volume, 8 years post-radiotherapy for VS and died within 2 months. All 8 malignancy patients died as a result of their malignancy or sequelae of treatment, the 1 survivor of 9 only just being post-operative although with good clearance on the first scan. For VS there are now 3 MPs of the primary tumor amongst 261 treated compared to 0/2049 VS not treated with radiation (*P* = .0015). Two of 32 (6.2%) patients having radiotherapy <18 years and 5/82 (6.1%) <25 years of age developed a malignancy/MP compared to only 4/184 (2.2%) after the 25th birthday (*P* = .14).

**Table 3.** Malignant or Presumed Malignant Disease Following Radiation-Based Treatment for Benign Disease

Case	Sex	Age at NF2 Diagnosis	NF2 Diagnosis VS	NF2 ­Analysis	Radiation Site/Type	Age RT	Malignant Progression/New ­Occurrence	Age Malignant Progression (MP)/new occurrence (NO)	Pathology	Delay Years	Age at Death *Death From Malignancy/RT	Ref
*New malignant tumor (potential induction cases 6009, 2373)*												
6009	F	13	Bilateral	frameshift deletion PV	stereotactic RT15Gy marginal dose, VS bilateral	15.6	Meningioma grade 3 initial meningioma within 1cm of VS treatment, not present at time of RT	32 NO	Yes	16.45	N/a <1 year from diagnosis	
2401	M	10	Bilateral	not identified	stereotactic RT, VS bilateral	21.7	MPNST -Biopsy-malignant myxoid tumor with INI1 loss at L4 not in RT field	30 MP	Yes	8.27	30.5*	
2373	F	52	Bilateral	splice acceptor site PV	stereotactic RT, VS bilateral 1.8 cm^3^ 14Gy marginal dose	58.1	Glioblastoma grade 4 brain stem	61 NO	Yes	2.94	62.1*	^7^
*Malignant progression/transformation*												
2393	M	21	Bilateral	not identified	stereotactic RT, VS bilateral 15Gy marginal dose to 3.3cm VS annual growth pre-RT 7mm	22.4	Schwannoma malignant progression no surgery. Initial shrinkage at 1 year to 2.8cm central necrosis. Representation 18 months later with 5.8cm VS moribund	25 MP	no	2.57	25.6*	^14^
1633	M	12	Bilateral	Splice site PV	stereotactic RT, VS unilateral 29.6mm VS 31 months before RT. 35.2mm 6 months before and 37.0mm on the date of SRS. Annualized growth rate 3mm	33.2	MPNST, malignant features on histology after removal of rapidly growing VS 7 years post-treatment	40.2	Yes	7.0	40.2* Died post-operatively from pneumonia.	
116	F	29	Bilateral	not identified	stereotactic RT, VS unilateral 15Gy marginal dose 3.9 cm^3^ (2.5cm diameter) from 1.8cm year before. Annual growth rate pre-RT 7mm	31.4	MPNST/Triton grade 3/malignant transformation of schwannoma 13.6 cm^3^ 4cm diameter: growth in 12 months 1.5cm diameter	34 MP	Yes	2.57	34.6*	^7^
1652	F	50	Right VS plus multiple meningiomas	not identified	Fractionated photon radiotherapy, meningioma	32.4	Meningioma grade 3, prior histology grade 1	47 MP	Yes	14.65	56.8*	^15^
186	F	18	Bilateral	not identified	Fractionated photon radiotherapy, spine C2-C4 3cm at RT	22.3	Ependymoma malignant progression spine Spread to involve brain stem (5cm superiorly) and most of spinal cord	23 MP	No	0.67	25.3*	
1462	F	16	Bilateral	Nonsense PV	Fractionated photon radiotherapy, spine T1-T3 3cm at RT	16.7	ependymoma malignant progression spine spread to C4-L1 with frontal lobe metastasis grade 3 ependymoma at post mortem	19 MP	Yes	2.28	20.5*	^15^
*Spinal progression after proton beam*												
60266	M	12	Bilateral	not identified	Proton beam spinal ependymoma 50Gy	14.2	N/a	N/a		N/a progressive quadriparesis and kyphoscoliosis. Death within 16 months	15.5* progressive ependymoma	

VS, Vestibular Schwannoma; RT, radiotherapy; MPNST, Malignant Peripheral Nerve Sheath tumor.

We also report a failure of proton beam radiotherapy for presumed intraspinal ependymoma aged 14 that had ongoing progression this is not included in the MP figures. The patient, with near-normal neurological function pretreatment, developed a progressive spastic quadriplegic within a few weeks of treatment and started to develop a spinal deformity which became a severe hyper-kyphosis with scoliosis, and respiratory function was compromised. Died within 15 months of treatment. Six of the fifteen spinal irradiation cases have died with 3 directly as a result of radiation treatment.

In the non-irradiated group, there were only 3 grade 3 meningiomas (0.27%) censored at diagnosis. There were 2 recent epithelioid sarcomas one affecting the spine which could have been of Schwann cell origin.^19^ The second was behind the knee and therefore not of CNS origin so was excluded from the main analysis. Both patients died from their sarcomas within 2 years aged 23 and 54 years. No unirradiated patient met the criteria for MP of VS or ependymoma. The only patient who died from slower ependymoma progression was a male who died aged 33 years, who had previously undergone unilateral VS Gamma Knife aged 16. He was not included in the MP group as he did not meet the growth criteria and the tumor was not in the radiation volume.

### Kaplan–Meier Analysis

**Figure 1.**
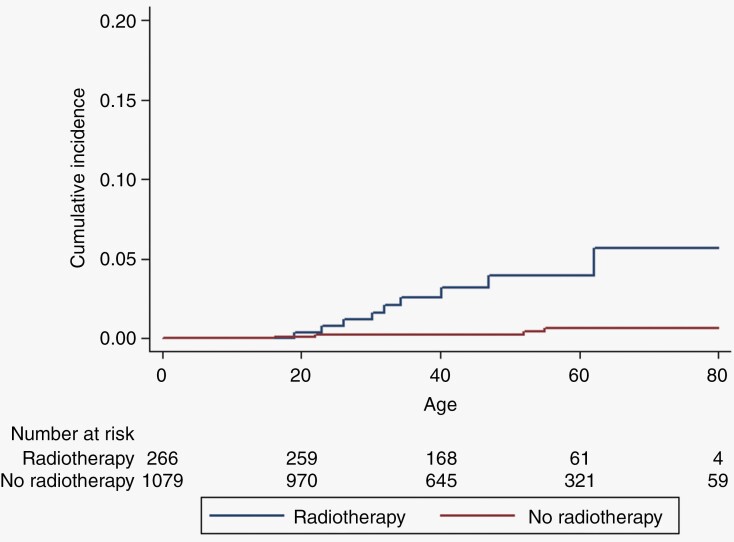
Kaplan–Meier for central nervous system malignancy post-radiation (9 radiotherapies, 4 no radiotherapy) using censor age central nervous system malignancy –Log-rank Chi-square = 19.54, *P* < .001, for those with radiotherapy (*n* = 266) and without radiotherapy (*n* = 1079).

**Figure 2.**
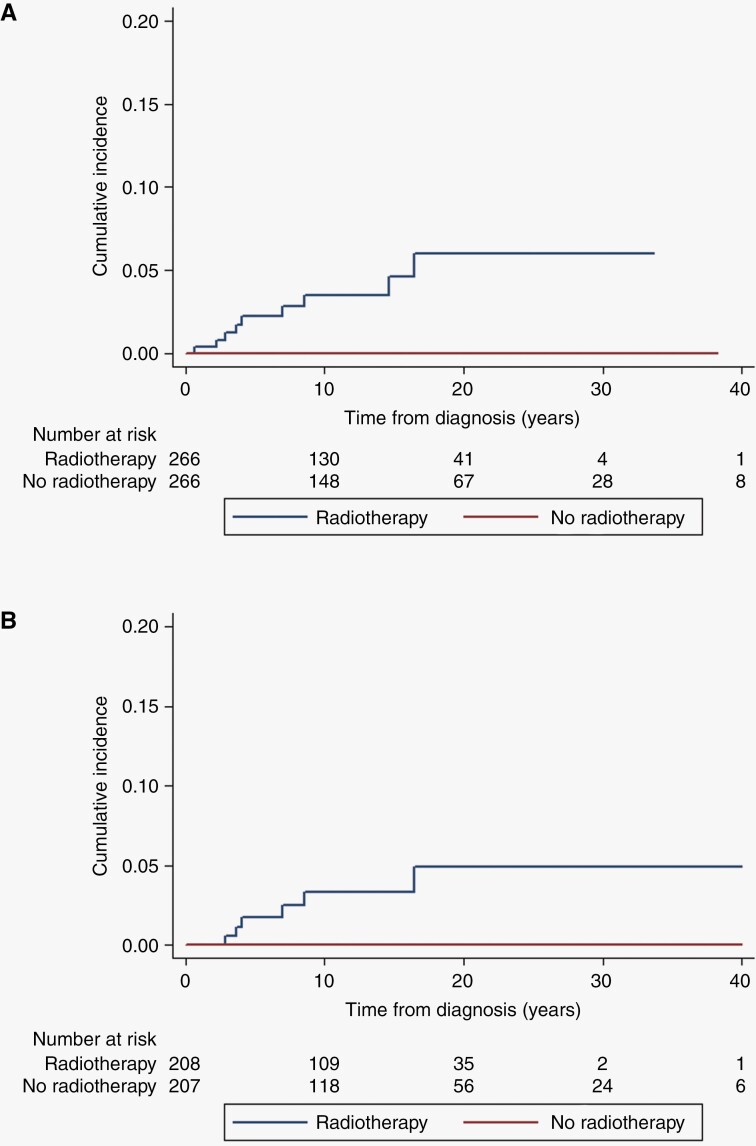
Kaplan–Meier for central nervous system malignancy (9 radiotherapies, 0 no radiotherapy) using matched cases for matched cases (*n* = 266 radiotherapy) and controls (*n* = 266 no radiotherapy) Log-rank Chi-square = 10.02, *P* = .0016.

**Figure 3.**
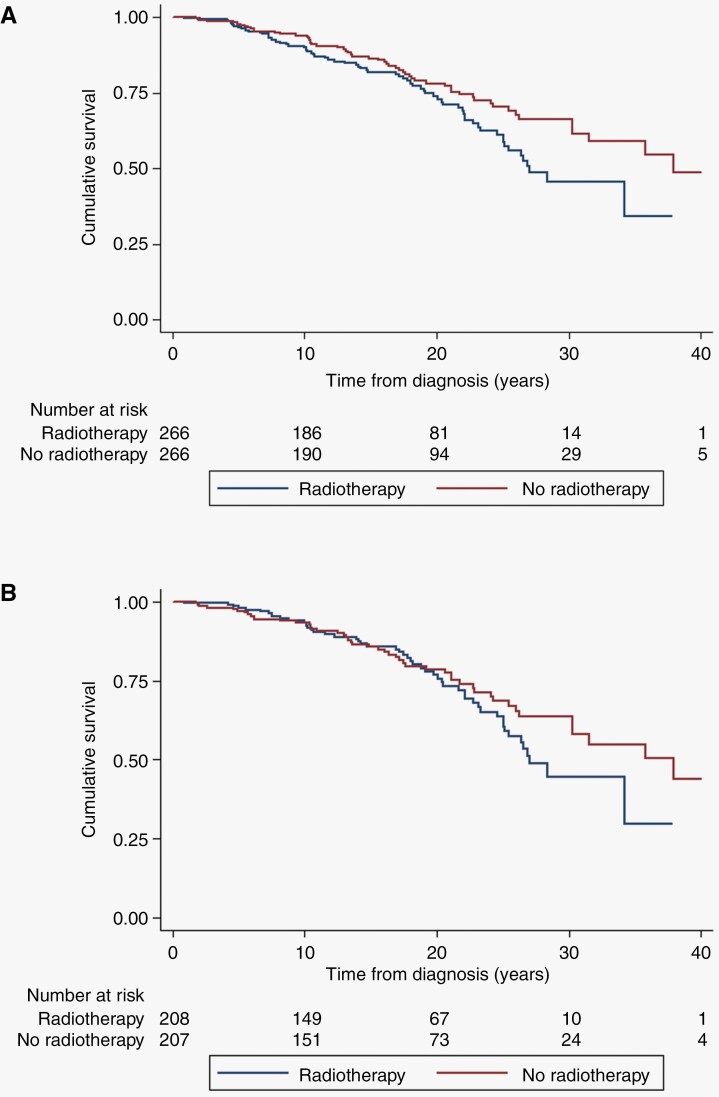
Kaplan–Meier for death (72 radiotherapies, 58 no radiotherapy) using follow-up from treatment for matched cases (*n* = 266 radiotherapy) and controls (*n* = 266 no radiotherapy). Log-rank Chi-square = 5.18, *P* < .023.

Lifetime incidence of CNS malignancy is shown in Figure 1 for the full cohort and shows a cumulative risk to age 70 years of 5.7% for irradiated patients compared to 0.7% in non-irradiated, equivalent to an absolute excess of 5% (*P* < .0001). Figure 2A shows a cumulative risk of 6% at 20 years post-radiotherapy for CNS malignancy (although 1/9 tumors were not in the radiation volume), this compares to zero in the matched set (*P* = .0016). For the VS matched set cumulative risk was 4.89% (95% CI = 2.06–11.4) for malignancy at 20 years-(Figure 2A; *P* = .01). If we exclude the spinal ependymoma without pathology this drops to 5.33% (95% CI = 2.34–11.87) risk of CNS malignancy at 70 years and a cumulative risk of 5.61% (95% CI = 2.63–11.8) at 20 years post-treatment (supplementary figures). Finally Figure 3 shows a reduced survival from treatment of the index tumor with survival at 30 years of only 45.6% (95% CI = 34.0–56.5) in irradiated cases, compared to 66.4% (95% CI = 57.3–74.0) in matched unirradiated controls. For VS this did not reach significance overall but survival at 30 years was 44.7% (31.2–57.3) compared to 63.8% (53.1–72.6) with the lower 95% CI for the unirradiated group not overlapping the RT group.

## Discussion

The present study represents by far the largest series of NF2 patients to the best of our knowledge that have undergone radiation treatment for the benign tumors that occur in the condition. The study shows a clearly increased incidence of malignancy/MP in irradiated CNS tumors with a cumulative risk of 6% at 20 years post-treatment. This is in contrast to unirradiated patients who have a lifetime risk of such malignancy of <1%. Many previous studies from centers that undertake radiation treatments comment on the likelihood that malignancy identified post-radiotherapy in individuals without prior biopsy of the treated tumor was in fact already malignant at the time of treatment.^7–10^ However, data from the present study and our previous work shows that such malignancy in particular MPNST and high-grade glioma is extremely uncommon in NF2 and has never been reliably reported in an NF2 VS without prior irradiation.^5,6^ Although the glioblastoma in the current report is rather early for an induced tumor from radiation after only 3 years, previous reports of glioblastoma in NF2 patients again only appear to have been after prior radiation treatment.^5,20^ The present report also highlights three malignant transformations/progressions of the target VS post-radiotherapy. None of these tumors had a malignant pattern of growth prior to treatment and had the typical initial enlargement from tumor swelling and then shrinkage before rapid enlargement occurred.^17^ Whilst induction of malignancy not in an index irradiated tumor typically occurs 7–20 years post-radiotherapy, malignant induction in the target VS typically occurs from 2 years onwards (2.6, 2.6, and 7 years in the current report). Survival from the malignancies in the current report is very poor in keeping with the literature and is likely to be a contributor to the poorer overall survival post-radiotherapy than in the matched non-irradiated patients.

There is also a concern about over-treating with radiation in childhood or early adulthood when it is known that risks are much higher for radiation induction.^21^ A nonsignificantly higher proportion of those irradiated under 25 years of age (6%) developed a malignancy/MP compared to older patients (2% *P*-.14). Radiation treatments in NF2 should therefore not be used as an early intervention in childhood when tumors are small and non-threatening. Tumors that are easily accessible surgically without any significant likelihood of deficit such as superficial meningiomas in particular should be offered surgery as the primary treatment option. Whilst radiation treatments will still have a place in treating tumors in NF2, particularly in older patients, in those with comorbidities and when tumors are inoperable, the current results suggest they should not be the first-line option for treatment of benign tumors, especially in children and young adults with NF2.

There is a particular concern about using radiotherapy for the usually indolent intraspinal ependymomas. These tumors are present often as small, frequently multiple foci on MRI and on the rare occasions they are biopsied or surgically removed, they are classified as ependymoma.^22,23^ Radiation therapy in 15 cases was associated with death within 4 years in 20% of cases. Of 15 patients who underwent spinal RT, 20% (3/15) died within 4 years all due to disease progression. Although in the United Kingdom, there has been a reluctance to undertake surgery for these tumors due to potential high morbidity, surgical outcomes when judicially planned can be very beneficial in the rare tumors that do require intervention.^23^ Bevacizumab can also be of benefit, especially for cystic ependymoma.^24^ One of the failed cases had undergone proton beam therapy. Whilst this form of radiation treatment is attractive in NF2 due to sharp fall-off in radiation dose around the target volume, there should also be reservations about using this form of radiotherapy in NF2, as 2/4 proton-treated cases have had poor outcomes, and the other 2 are too early in follow up to be certain.

The current study has some limitations. It was not feasible to match patients precisely on tumor burden or the size or growth rate of the index tumor prior to surgical/radiation/bevacizumab treatment. We did have tumor sizes for those with malignancy and none of the MPs were growing at a rate to suggest any likelihood of malignancy with 2 VS growing at 7mm and a third at only 3mm annually, very similar to VS treatments with bevacizumab-(Table 3).^4^ This might lead to speculation that those undergoing radiotherapy were more severely affected. In reality, the matched patient group was more likely to be severely affected as a higher proportion had full heterozygote and severe truncating pathogenic variants, which are associated with higher mortality^3^; far less had undiscovered variants, suggesting a higher proportion of irradiated patients had very low-level mosaicism and a better prognosis.^3,25^ Also, for the predominantly treated tumor, VS, there is a limitation because radiation is almost never used in the United Kingdom for tumors of >3.5cm (one of the malignant transformations was 37mm at treatment), whereas many surgical patients have tumors of greater than this size, including many of the matched control group. It is of note that all 3 of the malignancies after radiotherapy in VS were in tumors that were relatively large and faster growing so it is possible these would be more of a concern in any case for treating with radiation. Another potential bias is that radiotherapy patients may have had prior surgical or bevacizumab treatment. However, controls for the index tumor were also not selected on their first tumor treatment, and the delay from diagnosis to index tumor treatment was almost identical between cases and controls. Overall, our matching of patients is likely therefore to have selected a more severe phenotype in the non-irradiated group who then had better survival. In addition to the malignancy risk, radiation treatment is likely in a condition like NF2 to induce further meningiomas and schwannomas in the radiation volume, especially in children.^12,21^ This may contribute to increased death rate, although vascular events, unlike in NF1, do not seem to play a part (Table 1). We do not have volume of radiation for many patients so we are unable to assess a volume-treated risk assessment. We have not carried out detailed molecular analysis of the 8 potential radiation-induced malignancies, in part as 2 did not have material and 3 are no longer available. Previous reports on meningiomas have shown with RNA sequencing and methylation profiling, that *NF2* gene rearrangements were present in 12/31 of radiation-induced meningiomas,^26^ an observation previously unreported in sporadic meningioma. As our cohort only included 2 grade 3 meningiomas after radiation and these already had an underlying heterozygous *NF2* variant it is unlikely that we would have found an *NF2* rearrangement as a second hit. Combined losses of chromosomes 1p and 22q were also found to be common in radiation-induced meningiomas (16/18 cases) and overall, chromosomal aberrations were more complex than that observed in sporadic tumors.^26^ On the basis of 2 potentially radiation-induced tumors and three without radiation it is unlikely we would have been able to prove causality. No clear radiation-induced signals have been found in MPNST to the best of our knowledge other than from Nf1 mouse models.^27^

There are also major strengths to this study. It effectively represents a whole population study of NF2 patients with all NF2 patients being managed by just 4 centers.^7,28^ This is likely to represent extremely high ascertainment with no patients being totally lost to follow-up and deaths identified for all patients through the NHS registry system. This is in contrast to many radiation treatment centers which mainly discharge patients back to a local neurosurgical center for follow-up.

In summary, the present study represents the first major attempt to match radiation-treated NF2 patients to non-irradiated controls with comprehensive long-term follow-up. The study shows convincingly that there is a significant risk of malignancy/MP that needs to be discussed frankly with any NF2 patients, especially when that patient is young. In our view, radiation treatments should not be first-line treatment for benign tumors in NF2 patients especially given the future promise of drug treatments in the wake of the already proven benefits from bevacizumab.^4,29^ In an ideal world a randomized study of treatment interventions should be planned to assess survival and quality of life in NF2. The study also has implications for radiation treatments in other tumor predisposition syndromes, beyond NF1 where a high-risk of MPNST and high-grade glioma is already known after childhood irradiation,^30^ and especially for SMARCB1-related-schwannomatosis in which MPNST is already reported.^31^

## References

[1] Plotkin SR , Messiaen L, Legius E, et al. Revised diagnostic criteria and nomenclature for neurofibromatosis type 2 and schwannomatosis: An international consensus recommendation. Genet Med. 2022;24(9):1967–1977. 35674741 10.1016/j.gim.2022.05.007

[2] Evans DG , Huson SM, Donnai D, et al. A clinical study of type 2 neurofibromatosis. Q J Med. 1992;84(304):603–618. 1484939

[3] Hexter A , Jones A, Joe H, et al; English Specialist NF2 Research Group. Clinical and molecular predictors of mortality in neurofibromatosis 2: A UK national analysis of 1192 patients. J Med Genet. 2015;52(10):699–705. 26275417 10.1136/jmedgenet-2015-103290

[4] Forde C , King AT, Rutherford SA, et al. Disease course of neurofibromatosis type 2: a 30-year follow-up study of 353 patients seen at a single institution. Neuro Oncol. 2021;23(7):1113–1124. 33336705 10.1093/neuonc/noaa284 PMC8248850

[5] King AT , Rutherford SA, Hammerbeck-Ward C, et al. High-grade glioma is not a feature of neurofibromatosis type 2 in the unirradiated patient. Neurosurgery. 2018;83(2):193–196. 28973691 10.1093/neuros/nyx374

[6] King AT , Rutherford SA, Hammerbeck-Ward C, et al; English Specialist NF2 research group. Malignant peripheral nerve sheath tumors are not a feature of neurofibromatosis type 2 in the unirradiated patient. Neurosurgery. 2018;83(1):38–42. 28973692 10.1093/neuros/nyx368

[7] Rowe J , Grainger A, Walton L, Radatz M, Kemeny A. Safety of radiosurgery applied to conditions with abnormal tumor suppressor genes. Neurosurgery. 2007;60(5):860–864. 17460521 10.1227/01.NEU.0000255426.08926.95

[8] Mathieu D , Kondziolka D, Flickinger JC, et al. Stereotactic radiosurgery for vestibular schwannomas in patients with neurofibromatosis type 2: An analysis of tumor control, complications, and hearing preservation rates. Neurosurgery. 2007;60(3):460–468; discussion 468. discussion 46870. 17327790 10.1227/01.NEU.0000255340.26027.53

[9] Shinya Y , Hasegawa H, Shin M, et al. Long-term outcomes of stereotactic radiosurgery for vestibular schwannoma associated with neurofibromatosis type 2 in comparison to sporadic schwannoma. Cancers. 2019;11(10):1498. 31591325 10.3390/cancers11101498 PMC6827030

[10] Mohammed N , Hung YC, Xu Z, et al. Neurofibromatosis type 2-associated meningiomas: An international multicenter study of outcomes after Gamma Knife stereotactic radiosurgery. J Neurosurg. 2021;18(136):1–6. 10.3171/2020.12.JNS202814 34144518

[11] Seferis C , Torrens M, Paraskevopoulou C, Psichidis G. Malignant transformation in vestibular schwannoma: Report of a single case, literature search, and debate. J Neurosurg. 2014;121(suppl):160–166. 25434949 10.3171/2014.7.GKS141311

[12] Evans DG , Birch JM, Ramsden RT, Sharif S, Baser ME. Malignant transformation and new primary tumours after therapeutic radiation for benign disease: Substantial risks in certain tumour prone syndromes. J Med Genet. 2006;43(4):289–294. 16155191 10.1136/jmg.2005.036319 PMC2563223

[13] Evans DG , Baser ME, O’Reilly B, et al. Management of the patient and family with neurofibromatosis 2: A consensus conference statement. Br J Neurosurg. 2005;19(1):5–12. 16147576 10.1080/02688690500081206

[14] Rowe JG , Radatz M, Walton L, Kemeny AA. Stereotactic radiosurgery for type 2 neurofibromatosis acoustic neuromas: Patient selection and tumour size. Stereotact Funct Neurosurg. 2002;79(2):107–116. 12743432 10.1159/000070106

[15] Louis DN , Perry A, Wesseling P, et al. The 2021 WHO classification of tumors of the central nervous system: A summary. Neuro Oncol. 2021;23(8):1231–1251. 34185076 10.1093/neuonc/noab106 PMC8328013

[16] Evans DG , Hartley CL, Smith PT, et al; English Specialist NF research group. Incidence of mosaicism in 1055 de novo NF2 cases: Much higher than previous estimates with high utility of next-generation sequencing. Genet Med. 2020;22(1):53–59. 31273341 10.1038/s41436-019-0598-7

[17] McEvoy AW , Kitchen ND. Rapid enlargement of a vestibular schwannoma following gamma knife treatment. Minim Invasive Neurosurg. 2003;46(4):254–256. 14506573 10.1055/s-2003-42347

[18] Baser ME , Evans DG, Jackler RK, Sujansky E, Rubenstein A. Neurofibromatosis 2, radiosurgery and malignant nervous system tumours. Br J Cancer. 2000;82(4):998. 10732777 10.1054/bjoc.1999.1030 PMC2374414

[19] Linder C , Smith MJ, Bulman M, et al. Sarcoma in neurofibromatosis 2: Case report and review of the literature. Fam Cancer. 2019;18(1):97–100. 29761250 10.1007/s10689-018-0084-4

[20] Balasubramaniam A , Shannon P, Hodaie M, et al. Glioblastoma multiforme after stereotactic radiotherapy for acoustic neuroma: Case report and review of the literature. Neuro Oncol. 2007;9(4):447–453. 17704364 10.1215/15228517-2007-027 PMC1994102

[21] Ron E , Modan B, Boice JD, Jr, et al. Tumors of the brain and nervous system after radiotherapy in childhood. N Engl J Med. 1988;319(16):1033–1039. 3173432 10.1056/NEJM198810203191601

[22] Hagel C , Stemmer-Rachamimov AO, Bornemann A, et al. Clinical presentation, immunohistochemistry and electron microscopy indicate neurofibromatosis type 2-associated gliomas to be spinal ependymomas. Neuropathology. 2012;32(6):611–616. 22394059 10.1111/j.1440-1789.2012.01306.x

[23] Kalamarides M , Essayed W, Lejeune JP, et al. Spinal ependymomas in NF2: A surgical disease? J Neurooncol. 2018;136(3):605–611. 29188529 10.1007/s11060-017-2690-7

[24] Morris K , Afridi SK, Evans DG, et al. The response of spinal cord ependymomas to bevacizumab in patients with neurofibromatosis Type 2. J Neurosurg Spine. 2017;26(4):474–482. 27982762 10.3171/2016.8.SPINE16589

[25] Evans DG , Hartley CL, Smith PT, et al. Incidence of mosaicism in 1055 de novo NF2 cases: Much higher than previous estimates with high utility of next-generation sequencing. Genet Med. 2020;22(1):53–59. 31273341 10.1038/s41436-019-0598-7

[26] Agnihotri S , Suppiah S, Tonge PD, et al. Therapeutic radiation for childhood cancer drives structural aberrations of NF2 in meningiomas. Nat Commun. 2017;8(1):186. 10.1038/s41467-017-00174-7 PMC5543118 28775249

[27] Laurent D , Smith AE, Bessler WK, et al. Irradiation of Nf1 mutant mouse models of spinal plexiform neurofibromas drives pathologic progression and decreases survival. Neurooncol Adv. 2021;3(1):vdab063. 34131650 10.1093/noajnl/vdab063 PMC8193912

[28] Lloyd SK , Evans DG. Neurofibromatosis type 2 service delivery in England. Neurochirurgie. 2018;64(5):375–380. 26826883 10.1016/j.neuchi.2015.10.006

[29] Plotkin SR , Duda DG, Muzikansky A, et al. Multicenter, prospective, phase ii and biomarker study of high-dose bevacizumab as induction therapy in patients with neurofibromatosis type 2 and progressive vestibular schwannoma. J Clin Oncol. 2019;37(35):3446–3454. 31626572 10.1200/JCO.19.01367 PMC7098833

[30] Sharif S , Ferner R, Birch JM, et al. Second primary tumors in neurofibromatosis 1 patients treated for optic glioma: Substantial risks after radiotherapy. J Clin Oncol. 2006;24(16):2570–2575. 16735710 10.1200/JCO.2005.03.8349

[31] Evans DG , Huson SM, Birch JM. Malignant peripheral nerve sheath tumours in inherited disease. Clin Sarcoma Res. 2012;2(1):17. 23036231 10.1186/2045-3329-2-17 PMC3499223

